# Comparative Clinical Characteristics of Frail Older Adults in the Emergency Department: Long-Term Care Hospital versus Community Residence

**DOI:** 10.3390/jpm14101026

**Published:** 2024-09-26

**Authors:** Yunhyung Choi, Hosub Chung, Jiyeon Lim, Keon Kim, Sungjin Bae, Yoonhee Choi, Donghoon Lee

**Affiliations:** 1 Department of Emergency Medicine, Chung-Ang University Gwangmyeong Hospital, College of Medicine, Seoul, Chung-Ang University, 110, Deokan-Ro, Gwangmyeong-Si 14353, Republic of Korea; yunhyung0710@cau.ac.kr (Y.C.); hoshap@cau.ac.kr (H.C.); uzimuz85@gmail.com (S.B.); 2 Department of Emergency Medicine, College of Medicine, Ewha Womans University Seoul Hospital, Ewha Womans University, 260, Gonghang-Daero, Gangseo-Gu, Seoul 07804, Republic of Korea; jylim0923@ewha.ac.kr (J.L.); mikky5163@ewha.ac.kr (K.K.); 3 Department of Emergency Medicine, College of Medicine, Ewha Womans University Mokdong Hospital, Ewha Womans University, 1071, Anyangcheon-Ro, Yangcheon-Gu, Seoul 07985, Republic of Korea; unii@ewha.ac.kr

**Keywords:** geriatrics, long-term care, frail older patient, frailty

## Abstract

Background/objective: Older patients from long-term care hospitals (LTCHs) presenting to emergency departments (EDs) exhibit a higher prevalence of frailty than those from the community. However, no study has examined frailty in patients from LTCHs in the ED. This study compared frailty in older patients from LTCHs and the community. Methods: We retrospectively analyzed data from the EDs of three university hospitals between 1 August and 31 October 2023, involving 5908 patients (515 from LTCHs and 5393 from the community). The Korean version of the Clinical Frailty Scale (CFS-K) was used to assess individuals aged 65 and older. We compared clinical characteristics, frailty, length of stay (LOS), and diagnosis between patients from LTCHs (LTCH group) and the community (community group). Results: Among ED patients, 55.0% and 35.2% in the LTCH and the community groups, respectively, were frail (*p* < 0.001). Of these, 71.7% in the LTCH group were hospitalized compared with 53.1% in the community group (*p* = 0.001). The odds ratio for in-hospital mortality was 4.910 (95% CI 1.458–16.534, *p* = 0.010) for frail LTCH patients and 3.748 (95% CI 2.599–5.405, *p* < 0.001) for frail community patients, compared to non-frail patients. Conclusions: Patients from LTCHs with frailty had higher hospital admission rates and increased in-hospital mortality compared to those in the community at the same frailty level. This study offers essential insights into the characteristics of older patients in LTCHs for healthcare administrators and medical staff worldwide.

## 1. Introduction

It was not until the 19th century that efforts were made to distinguish older adults from the general adult category in medicine, leading to the development of geriatrics as a separate discipline [1]. Today, geriatrics is gaining increasing importance due to the rapid growth of the older adult population, driven by increased life expectancy [2]. The aging process is modifiable, and while it is possible to achieve longevity without severe disability [3], it is also possible to live a long life with functional limitations and disabilities [4].

The extension of life that people hope for does not imply a continuation of suffering; reducing unnecessary suffering is as important as delaying death. Therefore, it is unreasonable to treat the health of younger and older adults as equivalent, even within the broader adult category [5]. In response, geriatrics has made efforts to assess the health status of older adults, one of which is the concept of frailty [6].

The importance of frailty in geriatrics is increasingly recognized, and its application in both outpatient and inpatient settings has been actively discussed [7,8,9]. Emergency department (ED) studies focusing on frailty are also emerging [10,11,12], including those in the Republic of Korea. A notable distinction among older adults presenting to EDs in the Republic of Korea compared to other age groups is the difference in the location of ED presentation based on their health status [13]. Similar to patients in other age groups, in addition to residing in the community, older adults may be admitted to long-term care hospitals (LTCHs) for ongoing medical needs.

In the Republic of Korea, LTCHs are medical institutions staffed by doctors and nurses that primarily serve individuals who require prolonged hospitalization and treatment for geriatric conditions [13]. The number of patients aged 65 years and older admitted to LTCHs in the Republic of Korea has significantly increased, reaching 222,670 in 2021, more than double the number in 2010 [14]. Since LTCHs are specialized in chronic care, patients are transferred to higher-level medical institutions when their health condition deteriorates rapidly. As the number of LTCH inpatient admissions has increased, the number of patients transferred from LTCHs to EDs has also increased annually [15]. Among older patients presenting to EDs, those from LTCHs exhibit a higher prevalence of frailty than those from the community [16].

Previous studies have attempted to describe the association between frailty and prognosis in older patients in the ED. However, no study has examined frailty in patients in LTCHs in the ED. Therefore, this study aims to compare the type and clinical characteristics of frailty in LTCH- and community-based older patients.

## 2. Materials and Methods

### 2.1. Study Setting and Data Collection

This study retrospectively analyzed data collected from the National Emergency Department Information System (NEDIS) in the EDs of three university hospitals between 1 August and 31 October 2023. NEDIS, established in 2003 under Articles 15 and 17 of the Emergency Medical Service Act [17], is maintained by the National Emergency Medical Center (NEMC) to provide real-time clinical information from 404 emergency medical institutions nationwide. This system serves as a foundation for the development of an advanced emergency medical system and supports research and policy formulations related to emergency medical care [18].

NEDIS collects data based on the Order Communication System (OCS) or Electronic Medical Record (EMR) from a patient’s arrival at the ED until discharge or release from the hospital. The collected data are analyzed by NEMC the following year, and statistical reports are published. Each hospital can independently modify and verify the NEDIS data it enters. This study used NEDIS data from three EDs.

We excluded the following cases from the dataset in the following order: patients under 65 years of age; death on arrival at the hospital; out-of-hospital cardiac arrest; discharge against medical advice (DAMA) from the ED; non-medical visits, including cases where individuals visited solely for COVID-19 testing before admission; individuals visiting for the issuance of certificates; trauma; patients transferred from non-long-term care hospitals; and incomplete data.

### 2.2. Clinical Frailty Scale (CFS)

This study assessed frailty in older adults using the CFS, a scoring system proposed by Rockwood in 2005 [19] to measure fitness and frailty in individuals aged 65 years and older. The CFS is a frailty assessment tool that has been validated for predicting adverse outcomes in older adults. It is also useful in the ED setting due to its simplicity and high inter-rater reliability [7,10,11,12,20,21,22,23]. The CFS score ranges from 1 (very fit) to 9 (terminally ill), with higher scores indicating more frailty [24]. This study used the Korean version of the CFS (CFS-K) proposed by Ko et al. in 2021 [25]. Upon arrival at the ED, the patient or their caregiver was interviewed by a physician or nurse, who then assigned a CFS score. The CFS score reflected the patient’s frailty level as it was two weeks prior to the current visit. The CFS scores were recorded in the EMR. Frailty was categorized with a CFS score of 1–3 classified as non-frail, 4 as prefrail, and 5–9 as frail [19].

### 2.3. Variables and Outcome Measures

This study analyzed the following variables: age; sex; initial vital signs; mental status upon arrival; the Korean version of the Triage and Acuity Scale (KTAS) [26] based on the Canadian Emergency Department Triage and Acuity Scale [27,28]; frailty; ED clinical outcomes, including discharge, admission, and death (for calculating *p*-values); ED length of stay (LOS); final hospital clinical outcomes, including discharge, transfer, DAMA, and death; and hospital LOS. The admission category for ED clinical outcomes included admissions to a general ward (GW), intensive care unit (ICU), and transfers to LTCHs or other hospitals.

### 2.4. Statistical Analysis

Quantitative data are presented as mean ± standard deviation (SD), while qualitative variables are expressed as frequencies and percentages. Statistical significance was determined using Student’s *t*-test for quantitative variables and Fisher’s exact test or linear-by-linear association (chi-square test for trend) for qualitative variables, with a two-tailed *p*-value threshold of <0.05. We analyzed the predictive factors influencing in-hospital mortality and ICU admission using logistic regression. All statistical analyses were performed using Statistical Package for the Social Sciences version 26.0 (IBM, Armonk, NY, USA).

## 3. Results

**Figure 1:**
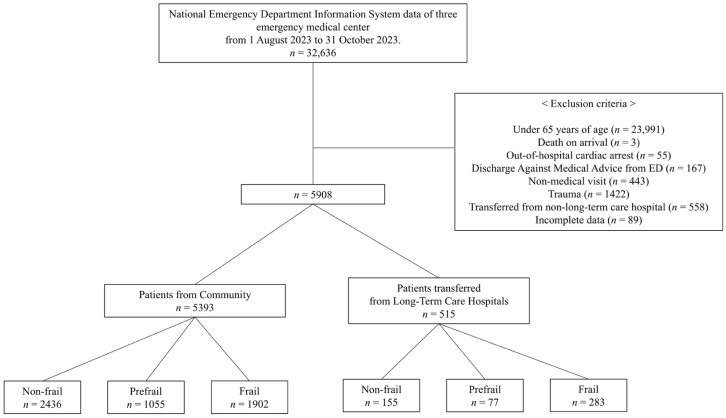
Study flowchart.

From 1 August to 31 October 2023, 32,636 patients attended the three EDs, and 5908 of them were finally included in the study after applying exclusion criteria. Of these, 515 were older patients transferred from LTCHs (LTCH group) and 5393 visited from the community (community group) (Figure 1).

### 3.1. Characteristics of Patients Transferred from Long-Term Care Hospitals and Those from the Community

There was a statistically significant difference between the mean age of patients in the LTCH and community groups (78.1 ± 7.7 vs. 76.6 ± 8.0, *p* < 0.001; Table 1). Notably, 86.0% and 92.5% of the patients in the LTCH and community groups, respectively, arrived at the ED in alert and conscious states (*p* < 0.001). Of the patients in the LTCH group, 55.0% were classified as frail compared to 35.2% in the community group (*p* < 0.001). The KTAS in the LTCH group was 0.8% level 1, 24.9% level 2, and 70.7% level 3, compared to 0.7% level 1, 14.8% level 2, and 70.9% level 3 in the community group (*p* < 0.001). In the LTCH group, 80.0% (*n* = 412, 49.5% GW, 22.5% ICU, 7.8% LTCH, and 0.2% other hospitals) were hospitalized compared to 43.4% (*n* = 2342, 31.9% GW, 10.0% ICU, 1.1% LTCH, and 0.2% other hospitals; *p* < 0.001) in the community group. ED LOS was 288.5 ± 238.6 min in the LTCH group and 228.3 ± 165.3 min in the community group (*p* < 0.001).

### 3.2. Comparison of Hospital Clinical Characteristics after Admission for Patients Admitted through the Emergency Department

Table 2 presents an analysis of patients admitted to the ED. In the LTCH group, 92.9%, 70.1%, and 71.7% of the non-frail, prefrail, and frail subgroups, respectively, were hospitalized (*p* = 0.001). Among the hospitalized patients, the LTCH and community groups had mortality rates of 8.7% and 7.8%, respectively (*p* < 0.001). The hospital LOS for patients in the LTCH group was significantly longer than that of those in the community group (15.2 ± 15.2 vs. 12.1 ± 12.9 days; *p* < 0.001).

### 3.3. Univariate Logistic Regression to Identify Predictors of In-Hospital Mortality and ICU Admission

Table 3 and Table 4 present the results of the logistic regression analysis performed to identify the predictors of in-hospital mortality and ICU admission, respectively, using variables found to be significantly different between the LTCH and community groups.

When patients were not alert, the odds ratios (ORs) for in-hospital mortality were 5.387 (95% confidence interval [CI] 2.563–11.322; *p* < 0.001) and 6.157 (95% CI 4.229–8.964; *p* < 0.001) in the LTCH and community groups, respectively. For frail patients, the ORs for in-hospital mortality were 4.910 (95% CI 1.458–16.534; *p* = 0.010) and 3.748 (95% CI 2.599–5.405; *p* < 0.010) in the LTCH and community groups, respectively.

Regarding ICU admission rates, when patients were not alert, the LTCH group had an OR of 4.871 (95% CI: 2.889–8.212; *p* < 0.001), and the community group had an OR of 9.828 (95% CI: 7.866–12.280; *p* < 0.001). When the patients were frail, the ICU admission rate in the community group had an OR of 1.903 (95% CI, 1.563–2.316; *p* < 0.001).

### 3.4. Comparison of Hospital Discharge Diagnosis between Patients from Long-Term Care Hospitals and the Community Based on the International Classification of Diseases, 10th Edition (ICD-10)

Table 5 summarizes the frequency analysis of the principal diagnoses of patients admitted to the ED based on ICD-10. Notably, 26.1% of the patients in the LTCH group had digestive system diseases, and 20.6% of those in the community group had circulatory system diseases.

## 4. Discussion

This study revealed that older patients in LTCHs exhibited higher rates of frailty and hospitalization. Furthermore, older patients with frailty demonstrated an increased risk of in-hospital mortality.

Frailty affects the health status of older adults and has been extensively studied. In the Republic of Korea, there are specialized medical institutions designated as LTCHs. These facilities are staffed by a multidisciplinary team of healthcare professionals, including doctors and nurses, and are equipped to provide long-term care and treatment for geriatric conditions [29]. Additionally, nursing homes for older adults exist; however, they differ from LTCHs in key ways. Unlike LTCHs, nursing homes do not have resident physicians and are primarily welfare facilities. They focus on assisting older patients with mobility issues due to geriatric diseases in their daily living activities rather than providing medical treatment [30]. As the older adult population continues to expand, the number of LTCH users is rapidly growing [31], highlighting the need for further research on frailty in LTCH-based patients. This study aimed to compare the clinical characteristics of patients in LTCHs and community-dwelling patients with similar levels of frailty.

Previous studies have demonstrated a correlation between higher frailty levels and increased mortality, hospitalization, and readmission rates [12,32,33]. Our study builds on this knowledge by revealing that, at equivalent levels of frailty, LTCH patients are more prone to hospitalization and that elevated frailty is associated with a higher risk of in-hospital mortality among LTCH patients. An analysis of the diagnoses of patients who died during their hospitalization (Table A1) revealed that the top five diagnoses were almost identical between the two groups, although their rankings differed.

In the LTCH group, frailty was not a significant predictor of ICU admission rates. This may be due to the ED physicians’ preconceived notions of LTCH patients [34,35,36,37], leading them to more aggressively admit patients to the ICU regardless of frailty. The LTCH group exhibited a higher proportion of patients with higher acuity (KTAS level 3 or higher: 96.3% in the LTCH group and 85.6% in the community group, *p* < 0.001), which may have also influenced the ED physician’s decision-making. In a study by Pulok et al., it was found that among patients with lower acuity, only those with higher frailty had increased mortality [38]. There are studies showing that higher acuity is associated with worse patient outcomes [39]. However, further research is needed to explore how frailty, in addition to acuity, impacts the prediction of patient prognosis.

A few limitations of this study need to be acknowledged. First, data from patients who were DAMA from the ED during the data collection phase were excluded due to their relatively small number. The specific reasons for DAMA in each case were not investigated, which may have affected the results. Second, this study analyzed data from the EDs of three university hospitals in the Seoul metropolitan area during a specific period. Selection bias may have occurred, limiting the generalizability of the study groups to the entire population. Third, there may be limitations arising from the relatively large size difference between the two groups. However, we attempted to minimize the limitations’ impact on the study design, analysis, and interpretation of the results. Further studies should not only elucidate the characteristics of the LTCH group but also provide guidelines for applying these findings in clinical practice and for the allocation of medical resources.

## 5. Conclusions

This study found that, among older patients presenting to the ED, admission rates for those in LTCH were higher than those for community dwellers, even for those with the same frailty status. Moreover, LTCH patients with frailty had a marked increase in in-hospital mortality. The ICU admission rates in LTCHs were not significantly associated with frailty, and further research is required to determine whether other factors are involved. This study offers essential insights into the characteristics of older patients in LTCHs for healthcare administrators and medical staff worldwide.

## Figures and Tables

**Table 1:** The characteristics of patients transferred from LTCHs ^1^ and patients from the community.

	Patients Transferred from LTCHs ^1^ *n* = 515	Patients from the Community *n* = 5393	*p*-Value
	*n* (%)	*n* (%)	
Age (mean ± SD ^2^)	78.1 ± 7.7	76.6 ± 8.0	<0.001
Male	249 (48.3)	2434 (45.1)	0.165
**Vital sign**			
SBP ^3^ (mmHg)	130.5 ± 27.8	140.1 ± 28.7	<0.001
DBP ^4^ (mmHg)	69.3 ± 15.5	74.9 ± 16.1	<0.001
PR ^5^ (/min)	89.3 ± 20.7	86.8 ± 20.4	0.008
RR ^6^ (/min)	20.2 ± 2.4	20.0 ± 2.2	0.099
BT ^7^ (°C)	37.0 ± 0.8	37.0 ± 0.9	0.965
SpO2 ^8^ (%)	96.3 ± 3.8	96.5 ± 3.7	0.360
**Mental status**			<0.001
Alert	443 (86.0)	4988 (92.5)	
Verbal responsive	42 (8.2)	210 (3.9)	
Painful responsive	27 (5.2)	171 (3.2)	
Unresponsive	3 (0.6)	24 (0.4)	
**KTAS** ^9^			<0.001
Level 1	4 (0.8)	36 (0.7)	
Level 2	128 (24.9)	799 (14.8)	
Level 3	364 (70.7)	3821 (70.9)	
Level 4	19 (3.7)	667 (12.4)	
Level 5	0 (0.0)	48 (2.0)	
**Frailty**			<0.001
Non-frail	155 (30.1)	2436 (45.2)	
Prefrail	77 (15.0)	1055 (19.6)	
Frail	283 (55.0)	1902 (35.2)	
**ED** ^10^ **clinical outcomes**			<0.001
Discharge	100 (19.4)	3040 (56.4)	
Admission	412 (80.0)	2342 (43.4)	
General ward admission	255 (49.5)	1721 (31.9)	
ICU ^11^ admission	116 (22.5)	551 (10.2)	
Transfer to LTCHs	40 (7.8)	61 (1.1)	
Transfer to other hospitals	1 (0.2)	9 (0.2)	
Death	3 (0.6)	11 (0.2)	
**ED LOS** ^12^ **(min)**			
mean ± SD	288.5 ± 238.6	228.3 ± 165.3	<0.001

^1^ LTCH = long-term care hospital, ^2^ SD = standard deviation, ^3^ SBP = systolic blood pressure, ^4^ DBP = diastolic blood pressure, ^5^ PR = pulse rate, ^6^ RR = respiratory rate, ^7^ BT = body temperature, ^8^ SpO2 = saturation of percutaneous oxygen, ^9^ KTAS = Korean Triage Acuity Scale, ^10^ ED = emergency department, ^11^ ICU = intensive care unit, ^12^ LOS = length of stay.

**Table 2:** Comparison of clinical characteristics of patients admitted to the emergency department.

	Admitted Patients Transferred from LTCHs ^1^ *n* = 371	Admitted Patients from the Community *n* = 2272	*p*-Value
	*n* (%)	*n* (%)	
**Hospital admission rate by each frailty level**	**/no. of frailty of patients visiting ED** **^2^** **(%)**	**/no. of frailty of patients visiting ED (%)**	0.001
Non-frail	114/155 (92.9)	842/2436 (34.6)	
Prefrail	54/77 (70.1)	420/1055 (39.8)	
Frail	203/283 (71.7)	1010/1902 (53.1)	
**Hospital final clinical outcomes**			<0.001
Discharge	222 (59.8)	1757 (77.3)	
Transfer	111 (29.9)	273 (12.0)	
Transfer to LTCH	89 (24.0)	165 (7.3)	
Transfer to other hospitals	22 (5.9)	108 (4.8)	
DAMA ^3^	5 (1.3)	65 (2.9)	
Death	33 (8.7)	177 (7.8)	
**Hospital LOS** ^4^ **(days)**			
mean ± SD ^5^	15.2 ± 15.2	12.1 ± 12.9	<0.001

^1^ LTCH = long-term care hospital, ^2^ ED = emergency department, ^3^ DAMA = discharge against medical advice, ^4^ LOS = length of stay, ^5^ SD = standard deviation.

**Table 3:** Univariate logistic regression analysis of in-hospital mortality predictors.

Variable	In-Hospital Mortality					
	Patients Transferred from LTCHs ^1^			Patients from the Community		
	OR ^2^ (95% CI ^3^)	B	*p*-Value	OR (95% CI)	B	*p*-Value
Age (years)	1.075 (1.025–1.126)	0.072	0.003	1.039 (1.016–1.062)	0.038	0.001
Sex; Male	1.304 (0.642–2.647)	0.265	0.463	0.696 (0.494–0.980)	−0.363	0.038
SBP ^4^ (mmHg)	0.992 (0.979–1.005)	−0.008	0.230	0.984 (0.976–0.993)	−0.016	<0.001
DBP ^5^ (mmHg)	0.982 (0.958–1.006)	−0.018	0.140	1.004 (0.989–1.019)	0.004	0.590
PR ^6^ (beats/min)	1.022 (1.006–1.038)	0.022	0.006	1.017 (1.009–1.025)	0.017	<0.001
RR ^7^ (breath/min)	1.088 (0.976–1.214)	0.085	0.129	1.099 (1.044–1.156)	0.094	<0.001
BT ^8^ (°C)	0.616 (0.386–0.983)	−0.484	0.042	0.847 (0.715–1.004)	−0.166	0.056
Altered mental status	5.387 (2.563–11.322)	1.684	<0.001	6.157 (4.229–8.964)	1.818	<0.001
**Frailty**						
Non-frail	1.00			1.00		
Prefrail	3.519 (0.818–15.129)	1.258	0.091	1.454 (0.877–2.409)	0.374	0.146
Frail	4.910 (1.458–16.534)	1.591	0.010	3.748 (2.599–5.405)	1.321	<0.001

^1^ LTCH = long-term care hospital, ^2^ OR = odds ratio, ^3^ CI = confidence interval, ^4^ SBP = systolic blood pressure, ^5^ DBP = diastolic blood pressure, ^6^ PR = pulse rate, ^7^ RR = respiratory rate, ^8^ BT = body temperature.

**Table 4:** Univariate logistic regression analysis of ICU admission predictors.

Variable	ICU ^1^ Admission					
	Patients Transferred from LTCHs ^2^			Patients from the Community		
	OR ^3^ (95% CI ^4^)	B	*p*-Value	OR (95% CI)	B	*p*-Value
Age (years)	1.021 (0.994–1.049)	0.021	0.130	1.022 (1.011–1.033)	0.021	<0.001
Sex; Male	1.139 (0.753–1.722)	0.130	0.539	1.365 (1.144–1.628)	0.311	0.001
SBP ^5^ (mmHg)	0.980 (0.972–0.988)	−0.020	<0.001	0.988 (0.985–0.991)	−0.012	<0.001
DBP ^6^ (mmHg)	0.972 (0.958–0.987)	−0.028	<0.001	0.987 (0.981–0.992)	−0.013	<0.001
PR ^7^ (beats/min)	1.017 (1.007–1.027)	0.017	0.001	1.016 (1.012–1.020)	0.016	<0.001
RR ^8^ (breath/min)	1.149 (1.049–1.258)	0.139	0.003	1.178 (1.137–1.220)	0.164	<0.001
BT ^9^ (°C)	1.071 (0.829–1.385)	0.069	0.599	1.055 (0.959–1.161)	0.053	0.273
Altered mental status	4.871 (2.889–8.212)	1.583	<0.001	9.828 (7.866–12.280)	2.285	<0.001
**Frailty**						
Non-frail	1.00			1.00		
Prefrail	0.802 (0.384–1.675)	−0.220	0.557	1.163 (0.899–1.505)	0.151	0.250
Frail	1.567 (0.967–2.538)	0.449	0.068	1.903 (1.563–2.316)	0.643	<0.001

^1^ ICU = intensive care unit, ^2^ LTCH = long-term care hospital, ^3^ OR = odds ratio, ^4^ CI = confidence interval, ^5^ SBP = systolic blood pressure, ^6^ DBP = diastolic blood pressure, ^7^ PR = pulse rate, ^8^ RR = respiratory rate, ^9^ BT = body temperature.

**Table 5:** Comparison of discharge diagnosis at the hospital between patients from LTCHs and the community based on ICD-10.

Patients Transferred from LTCHs ^1^ *n* = 371		Patients from the Community *n* = 2272	
ICD-10 ^2^	*n* (%)	ICD-10	*n* (%)
Diseases of the digestive system	97 (26.1)	Diseases of the circulatory system	467 (20.6)
Diseases of the circulatory system	77 (20.8)	Diseases of the digestive system	359 (15.8)
Diseases of the respiratory system	48 (12.9)	Neoplasms	264 (11.6)
Neoplasms	35 (9.4)	Diseases of the respiratory system	245 (10.8)
Diseases of the genitourinary system	30 (8.1)	Diseases of the genitourinary system	208 (9.2)

^1^ LTCH = long-term care hospital, ^2^ ICD-10 = International Classification of Diseases 10th edition.

## Data Availability

The datasets used in this study are available from the corresponding author upon request.

## Appendix A

**Table A1:** High-frequency discharge diagnoses for patients who died after hospitalization based on ICD-10.

Patients Transferred from LTCH ^1^ *n* = 33		Patients from the Community *n* = 177	
ICD-10 ^2^	*n* (%)	ICD-10	*n* (%)
Diseases of the circulatory system	7 (21.2)	Diseases of the circulatory system	41 (23.2)
Diseases of the respiratory system	7 (21.2)	Diseases of the respiratory system	38 (21.5)
Neoplasm	5 (15.2)	Neoplasm	33 (18.6)
Symptoms, signs, and abnormal clinical and laboratory findings, not elsewhere classified	5 (15.2)	Codes for special purpose	17 (9.6)
Certain infectious and parasitic diseases	3 (9.1)	Symptoms, signs, and abnormal clinical and laboratory findings, not elsewhere classified	14 (7.9)

^1^ LTCH = long-term care hospital, ^2^ ICD-10 = International Classification of Diseases 10th edition.
